# Supplementary material for: A fully integrated, standalone stretchable device platform with in-sensor adaptive machine learning for rehabilitation
Source: Nat Commun. 2023 Nov 27;14:7769. doi: 10.1038/s41467-023-43664-7 (PMC10682047; doi:10.1038/s41467-023-43664-7)
Supplement: Supplementary file 1 — Supplementary information [file 41467_2023_43664_MOESM1_ESM.pdf]

A fully integrated, standalone stretchable device platform with in-sensor adaptive machine learning for rehabilitation

Hongcheng Xu[1], Weihao Zheng[1], Yang Zhang[2], Daqing Zhao[3], Lu Wang[3], Yunlong Zhao[4], Weidong Wang[1], Yangbo Yuan[1], Ji Zhang[1], Zimin Huo[1], Yuejiao Wang[5], Ningjuan Zhao[1], Yuxin Qin[1], Ke Liu[1], Ruida Xi[1], Gang Chen[1], Haiyan Zhang[1], Chu Tang[6], Junyu Yan[1], Qi Ge[7], Huanyu Cheng[8], Yang Lu[9], Libo Gao[4]

[1]School of Mechano-Electronic Engineering, Xidian University, Xian 710071, China

[2]Department of Medical Electronics, School of Biomedical Engineering, Air Force Medical University, Xi’an 710032, China

[3]Department of Otolaryngology-Head and Neck Surgery, The Second Affiliated Hospital of Air Force Medical University, Xi'an 710032, China

[4]Pen-Tung Sah Institute of Micro-Nano Science and Technology, Xiamen University, Xiamen, 361102, China

[5]Applied Mechanics Laboratory, Department of Engineering Mechanics, Tsinghua University, Beijing 100084, P.R. China

[6]Engineering Research Center of Molecular and Neuro Imaging, Ministry of Education, School of Life Science and Technology, Xidian University, Xi’an, Shaanxi 710126, China

[7]Department of Mechanical and Energy Engineering, Southern University of Science and Technology, Shenzhen 518055, China.

[8]Department of Engineering Science and Mechanics, The Pennsylvania State University, University Park, PA 16802, USA

[9]Department of Mechanical Engineering, The University of Hong Kong, Pokfulam,

Hong Kong 999077, Hong Kong SAR

Correspondence should be addressed to W.W. (E-mail: wangwd@mail.xidian.edu.cn); H.C. (E-mail: Huanyu.Cheng@psu.edu); L.Y. (E-mail: ylu1@hku.hk); and L.G. (E-mail: lbgao@xmu.edu.cn).

Note 1: Offline sample collection for testing convolutional neural network.

The overall trained data were collected from five healthy human subjects (i.e., four males and one female) who were asked to perform the designed behavior tasks. To validate the convolution neural network (CNN)-based network model and evaluate signal quality, the 2D-SFE was used to analyze the laryngeal activities with three performing states from two new human subjects (not used for the above training). These states were to mimic the physical conditions and special movements/actions of patients. Three performing states include: 1) random interference to the biophysical behaviors (e.g., drinking water while coughing and talking with others while swallowing); 2) all activities with random chewing; 3) tested actions with nodding.

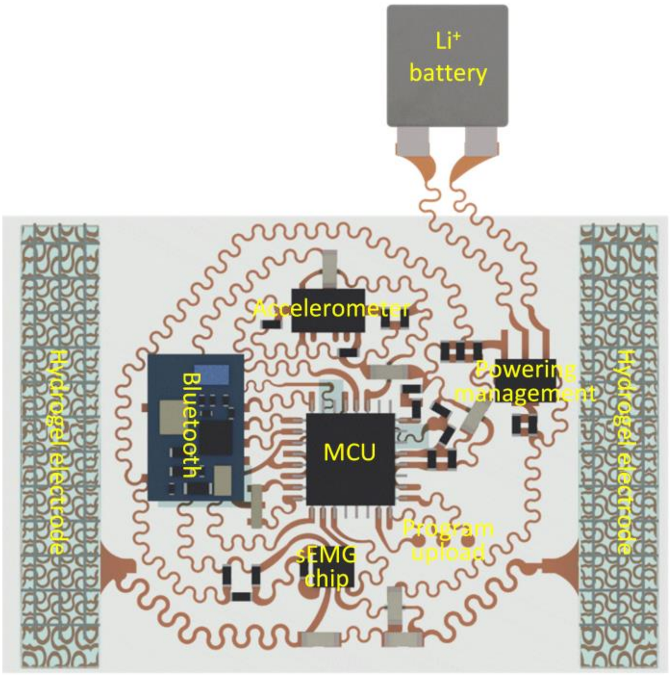

Fig. S1. Schematic showing the device components in the laryngeal patch.

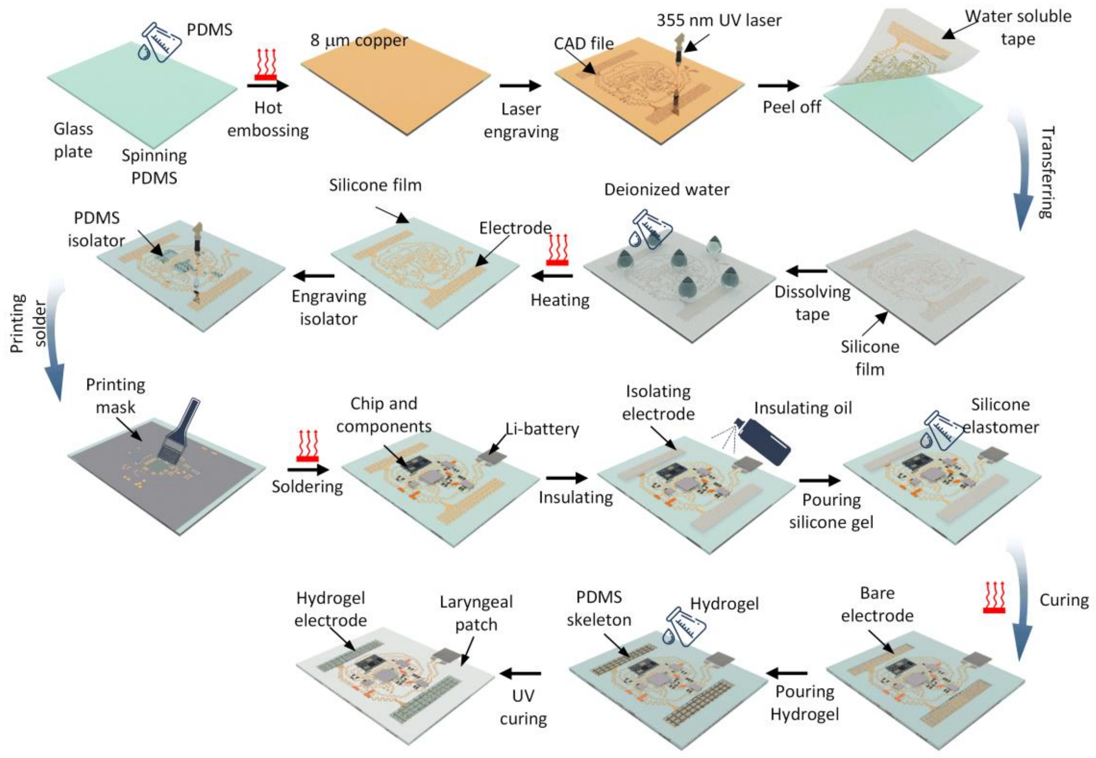

Fig. S2. Fabrication process of the laryngeal patch.

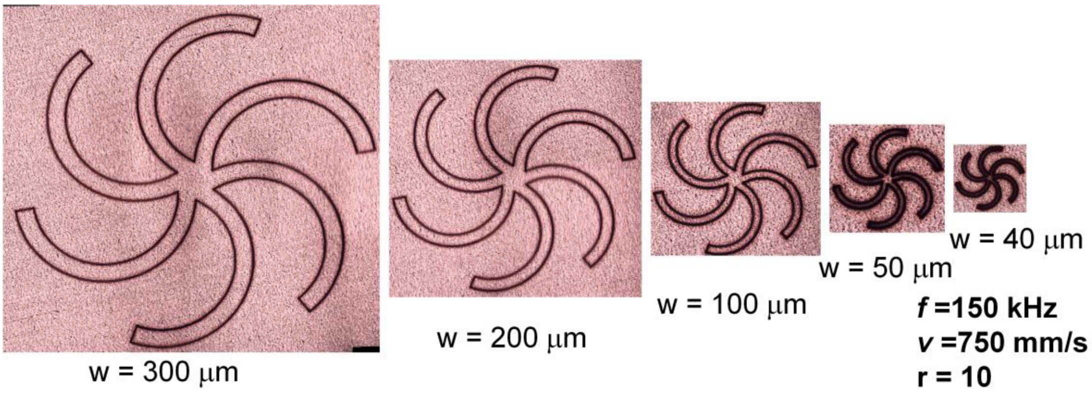

Fig. S3. Optical images of the serpentine structures with different line widths under 10 repeated continuous laser engraving steps.

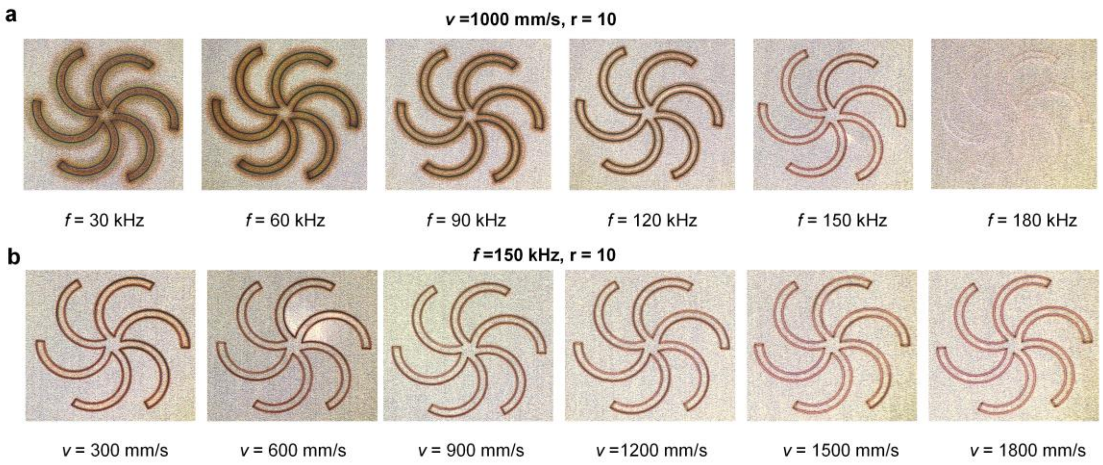

Fig. S4. Optical images of the serpentine structures under continuous laser engraving with different a pulse frequencies and **b** laser speeds**.**

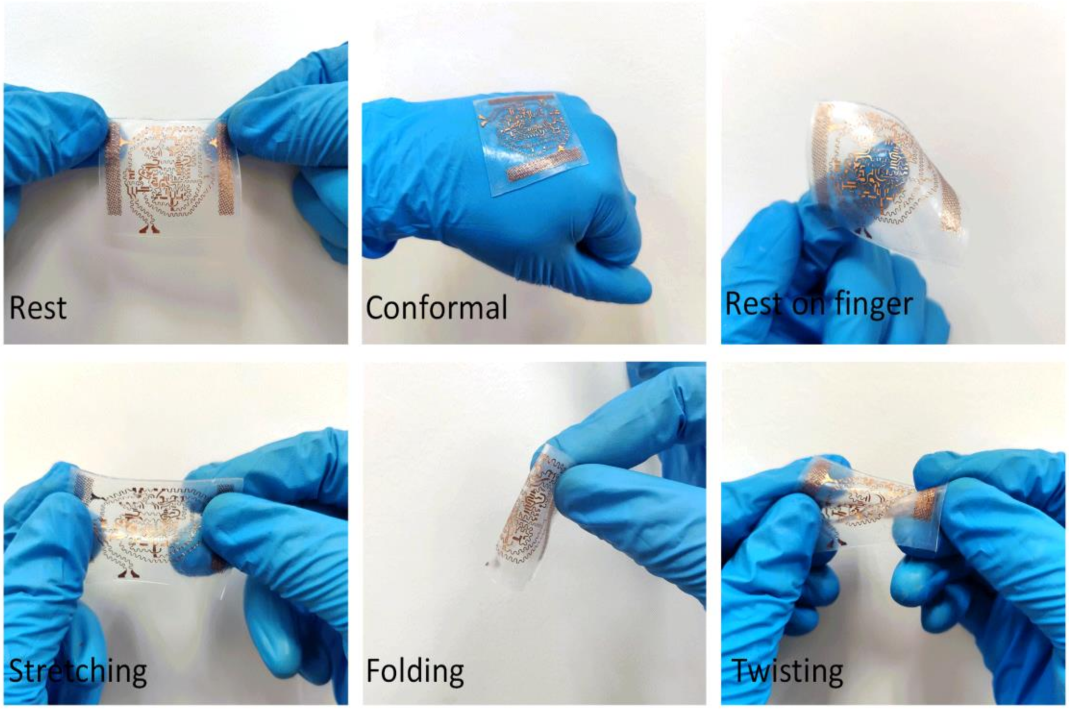

Fig. S5. Optical images of the stretchable serpentine trace on an ultra-soft elastomer before and after conformal contact on the back of the hand, resting on the fingertip, uniaxial stretching, folding, and twisting.

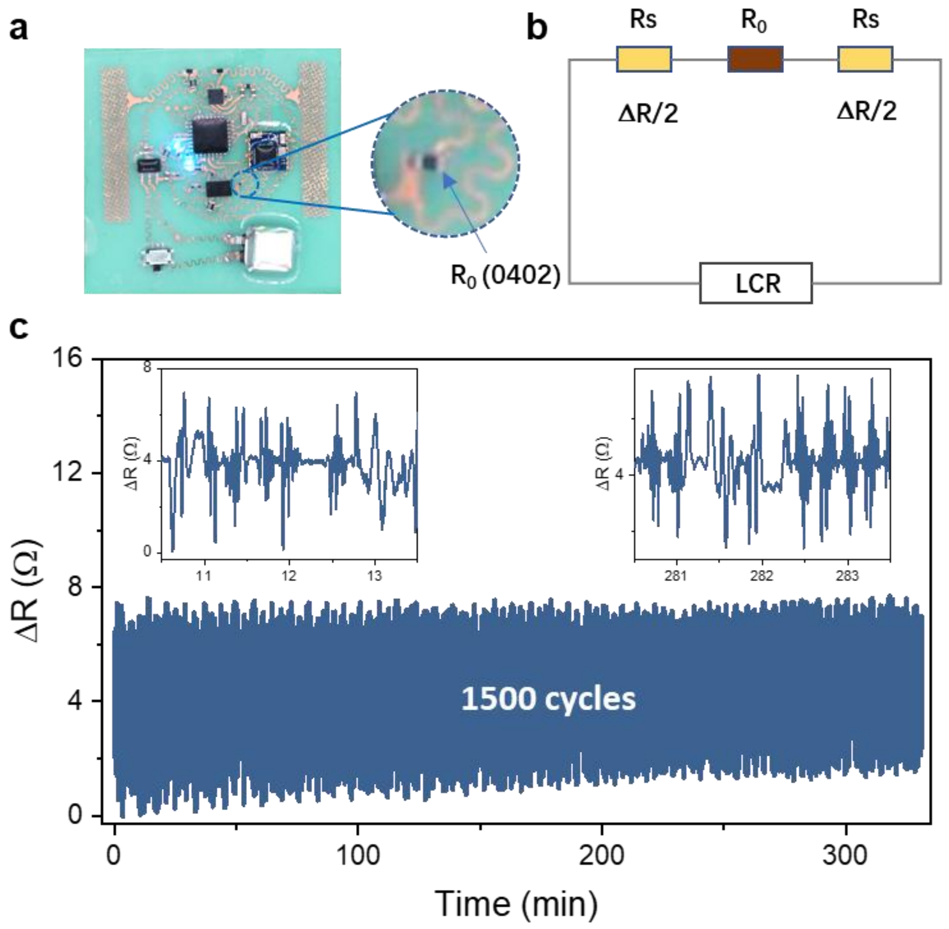

Fig. S6. Stretching test at the representative soldering region. a Optical image of the integrated device with the representative soldering region connected to a packaged resister (1 kΩ) shown in the inset. **b** Equivalent circuit of the soldering region. **c** Long-term durability test over 1500 cycles for stretching of 30%.

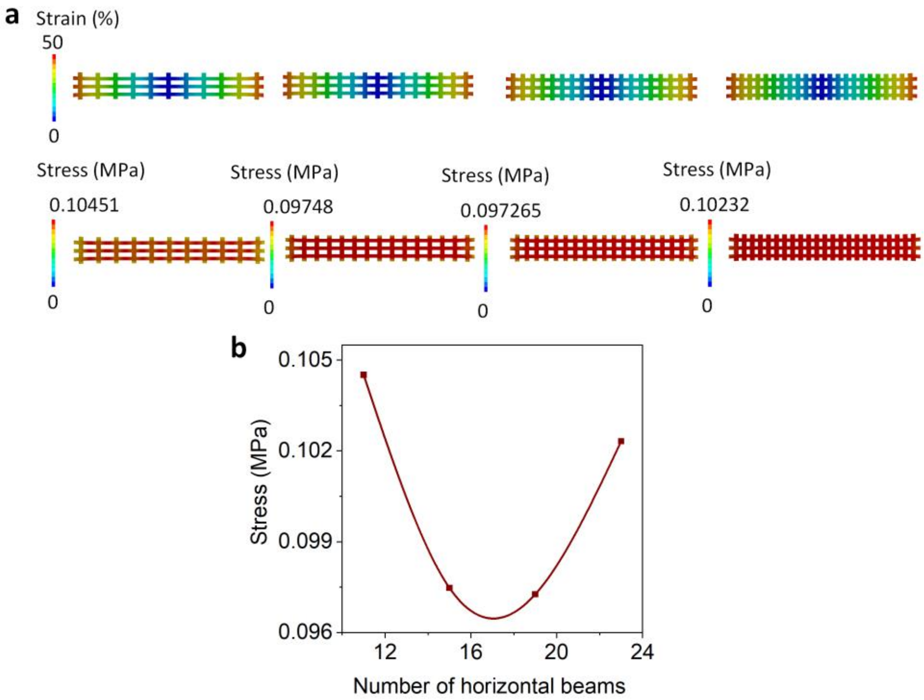

Fig. S7. Finite element analysis of the PDMS mesh structures with different numbers of vertical beams under the uniaxial tensile strain of 50%. a Strain (top) and stress distributions (bottom) of the mesh structure with 11, 15, 19, and 23 vertical beams (from left to right). **b** Maximum principal stress as a function of the number of vertical beams.

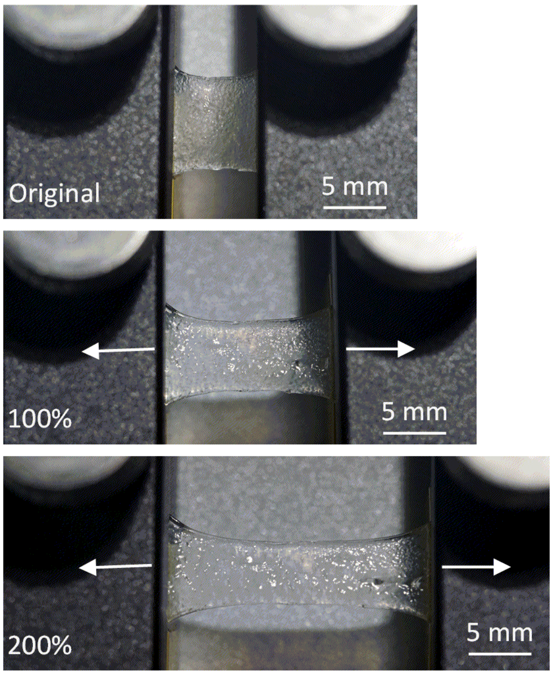

Fig. S8. Optical images of the modified hydrogel under the uniaxial tensile strain of 0, 100, and 200%.

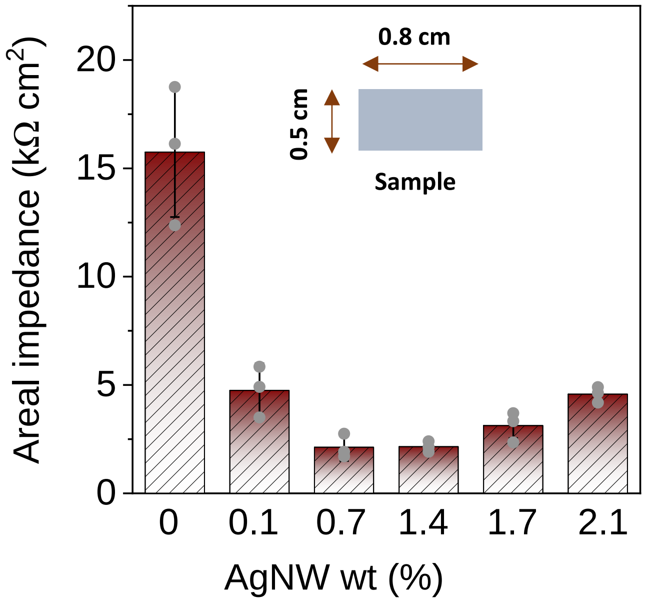

Fig. S9. Sheet resistance of the modified hydrogel with varying weight concentrations of AgNWs from 0 to 2.1%.

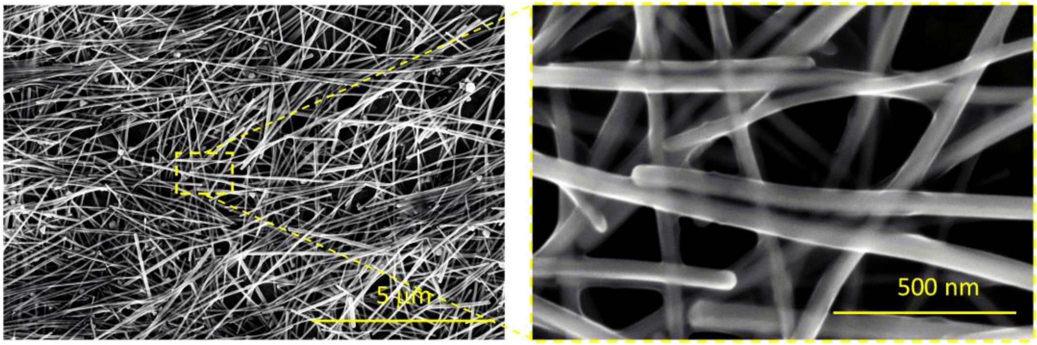

Fig. S10. Scanning electron microscope (SEM) image of Ag NWs (left) and its zoomed-in view (right).

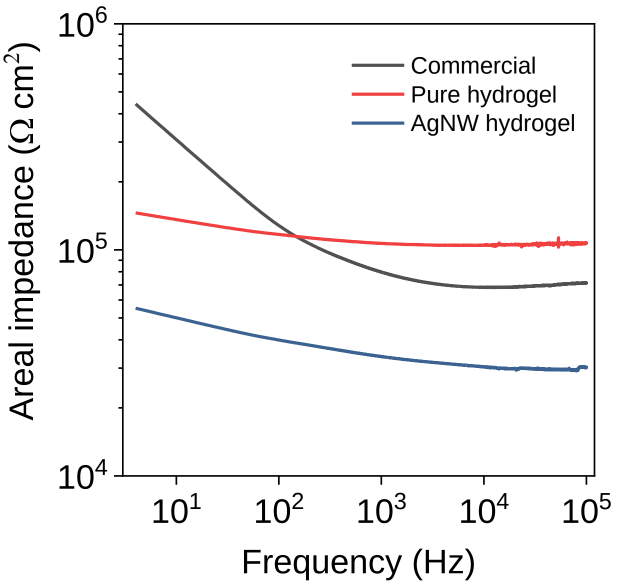

Fig. S11. Comparison in the areal contact impedance of electrodes based on the commercial gel, pure hydrogel, and modified hydrogel (with AgNWs of 0.7%)

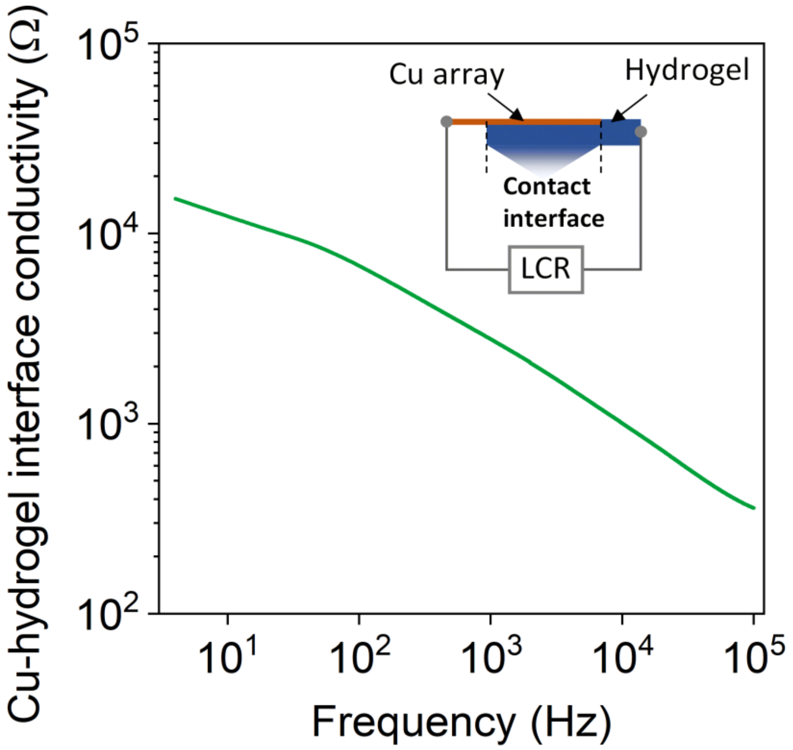

Fig. S12. **Resistance at the interface between the Cu-mesh electrode and the composite hydrogel over the frequency range from 4 Hz to 100 kHz, demonstrating high conductivity at the interface.**

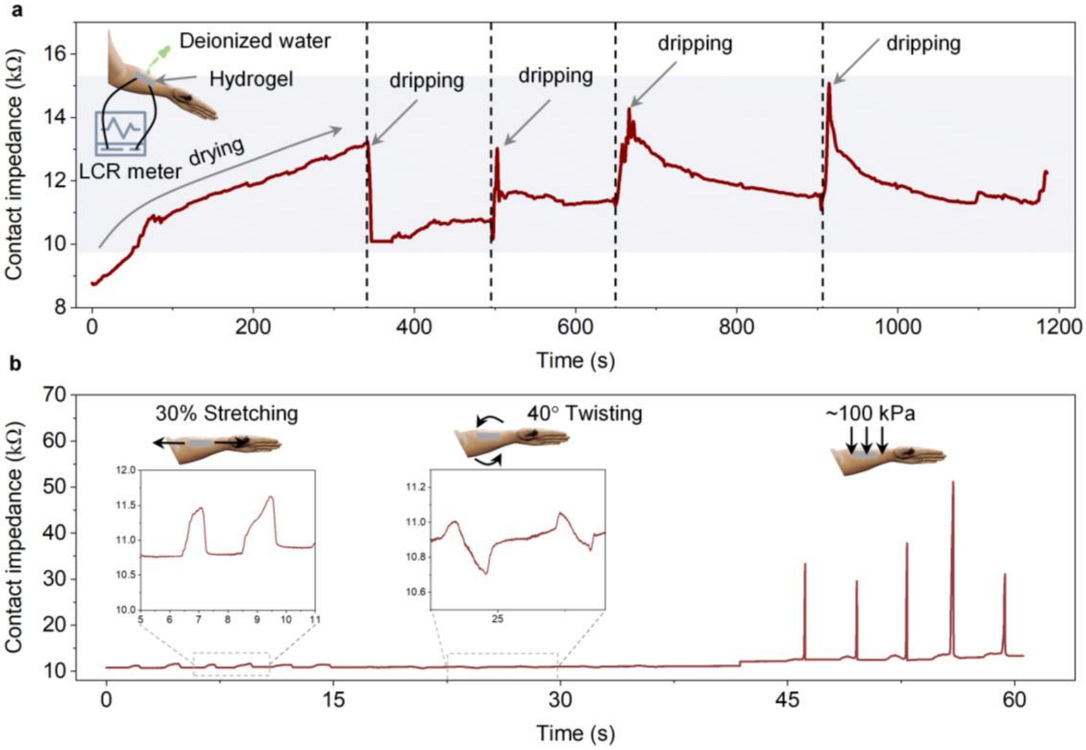

Fig. S13. Contact impedance measured with the modified hydrogel (size: 0.5×1.5 cm^2) placed on the forearm under a cyclic natural drying and dripping with deionized water and **b** continuous deformation.

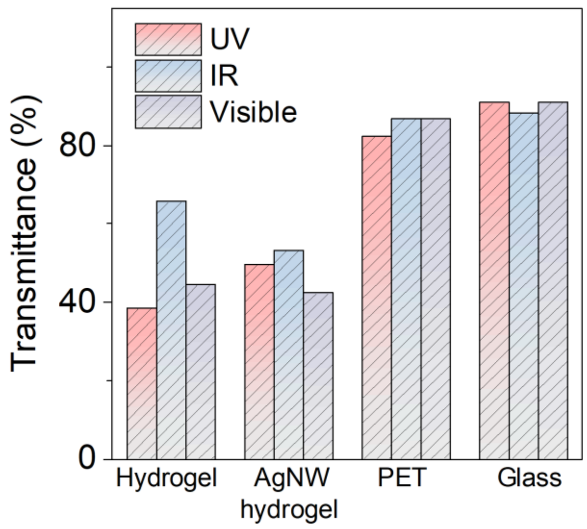

Fig. S14. UV, infrared (IR), and visible light transmittance of the pure hydrogel, modified hydrogel, PET film, and glass plate (thickness of 1 mm).

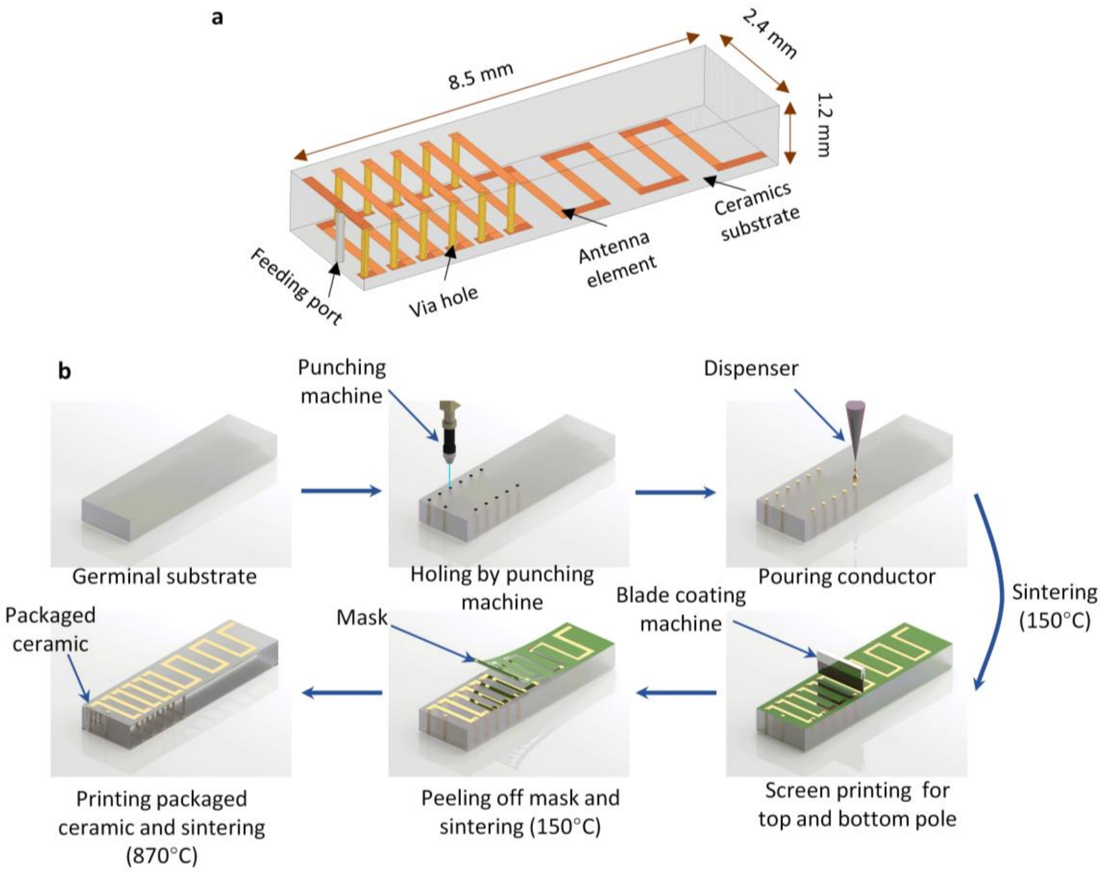

Fig. S15. Fabrication of the low-temperature co-fired ceramic (LTCC) antenna. a Design and **b** fabrication process of the antenna.

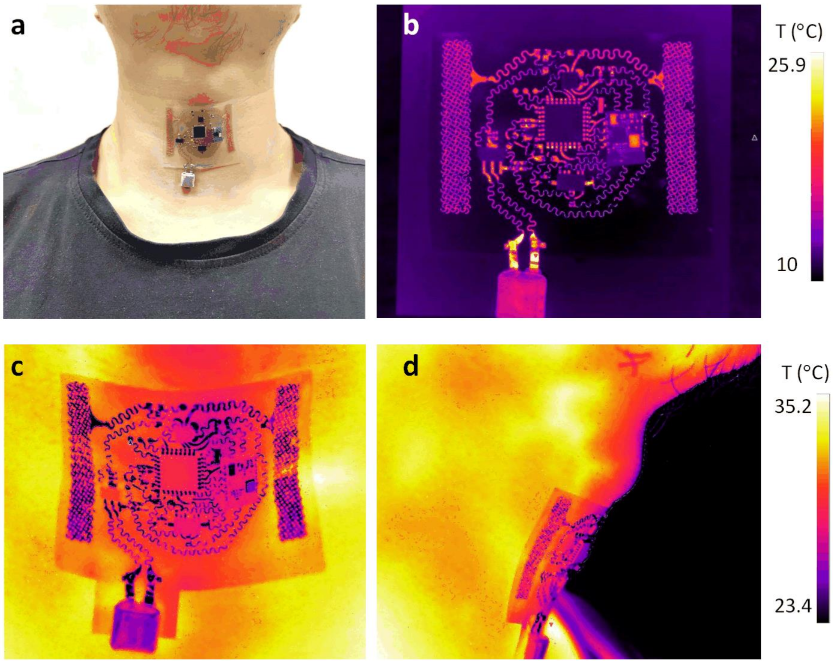

Fig. S16. Optical and thermal images of the patch on the laryngeal skin. a Optical image of the patch on the skin. Thermal images to show the comparison in the temperature distribution of the powered patch on **b** a cooled platform (10°C) and on the skin in the **c** front and **d** side views.

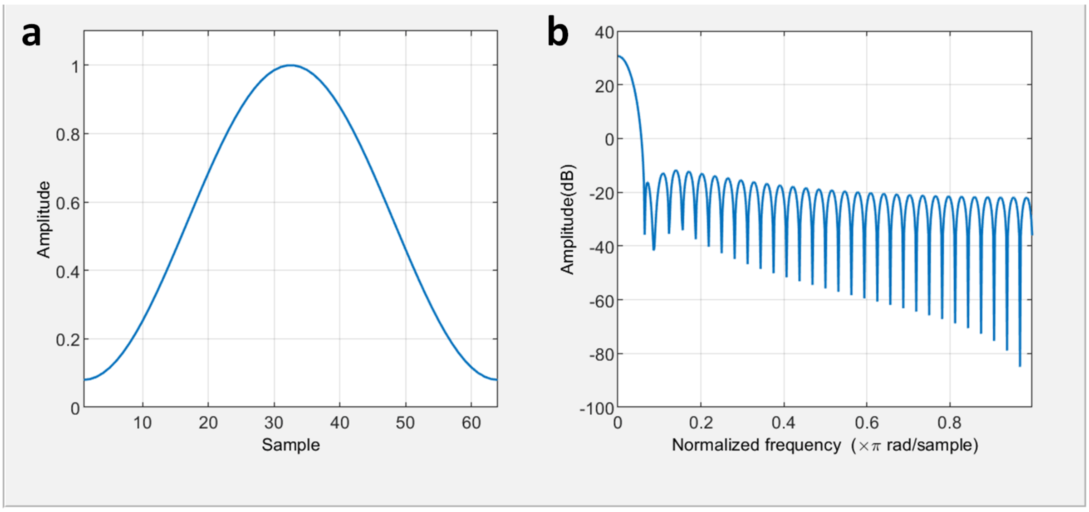

Fig. S17. Hamming windows set for the acquisition of the power spectrum density and signal noise ratio (SNR) in a time and **b** frequency domains (window length of 64).

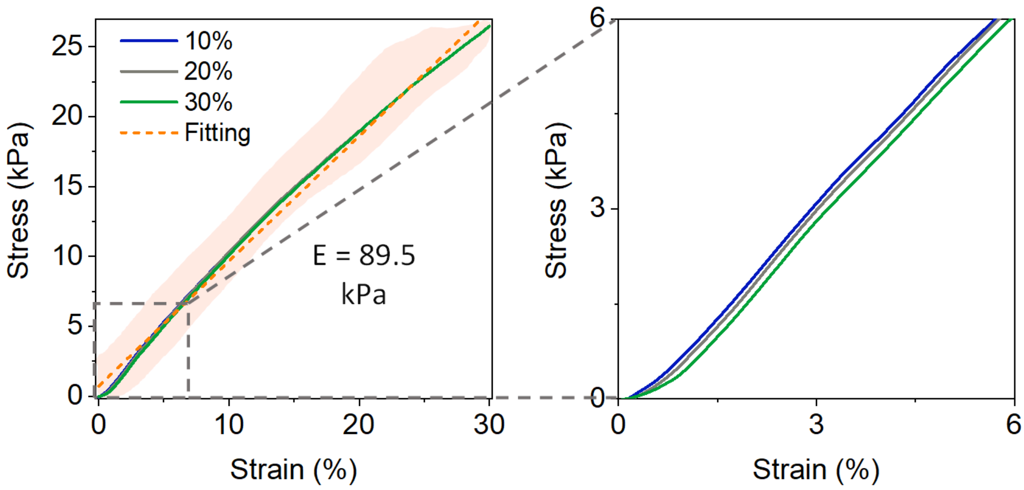

Fig. S18. Stress-strain curves of the stretchable patch under the uniaxial tensile strain up to 30 % (left) and its zoomed-in view (right).

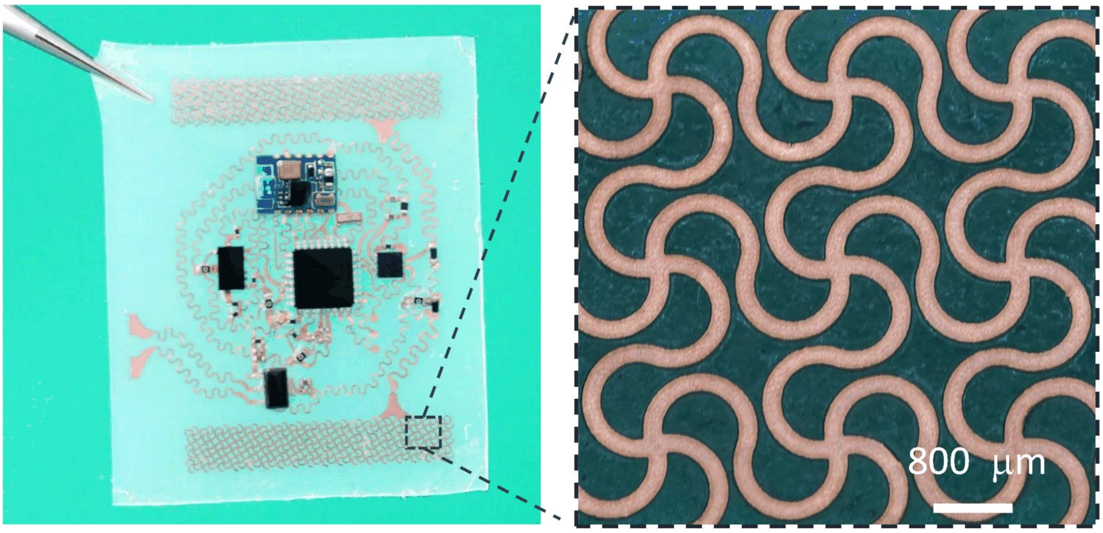

Fig. S19. Optical images of the fabricated laryngeal patch (left) and its zoomed-in view showing the serpentine traces (right).

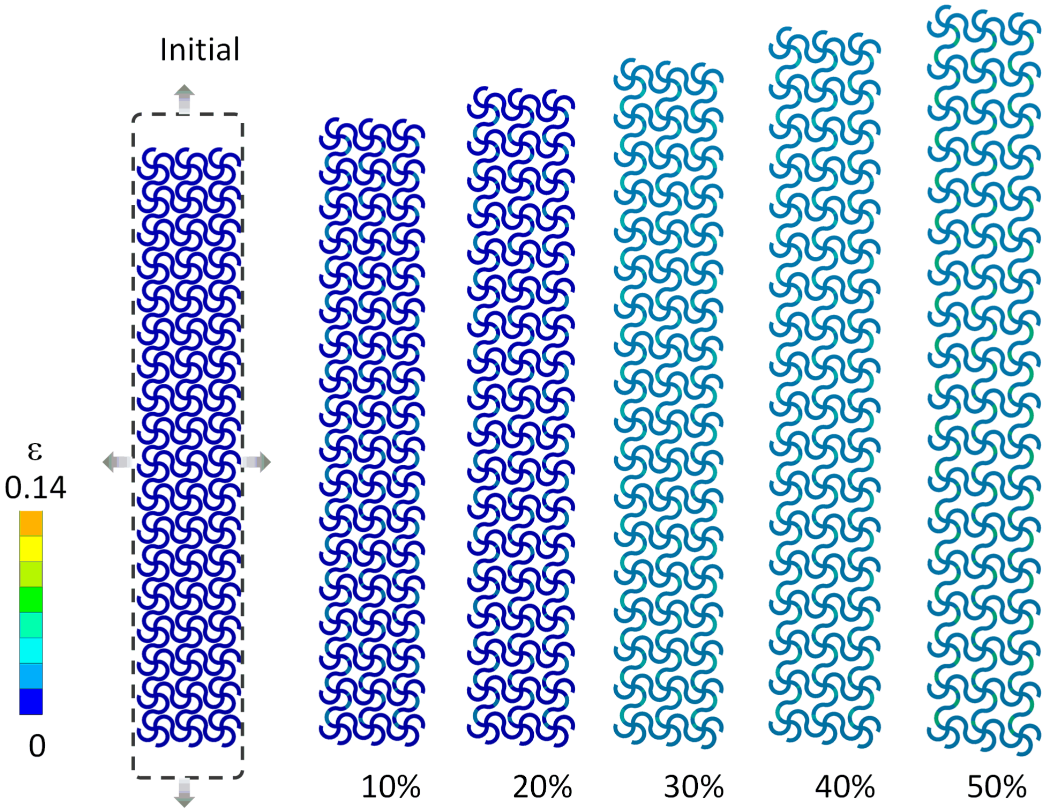

Fig. S20. Strain distribution in the serpentine copper electrode under the biaxial tensile strain of 0, 10, 20, 30, 40, and 50%.

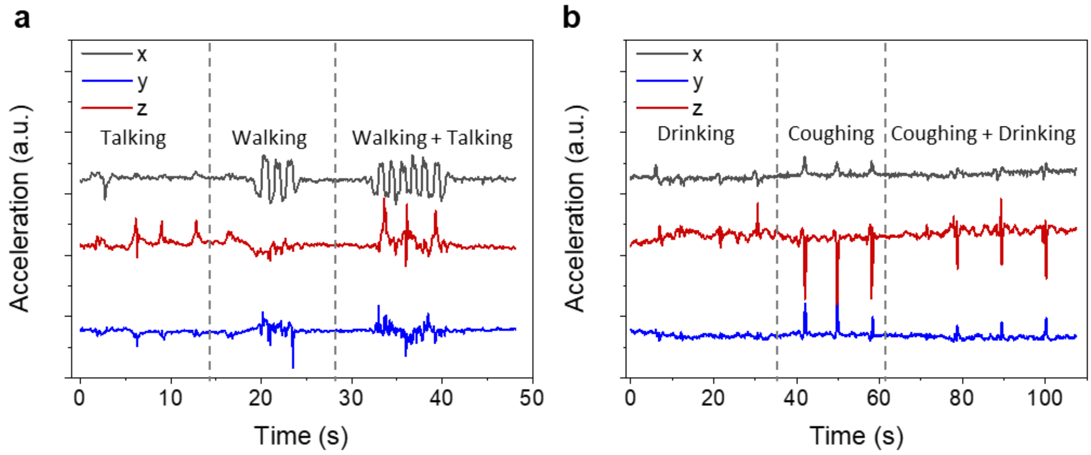

Fig. S21. Triaxial accelerations captured by the stretchable patch at the throat to identify a talking while walking and **b** drinking water while coughing.

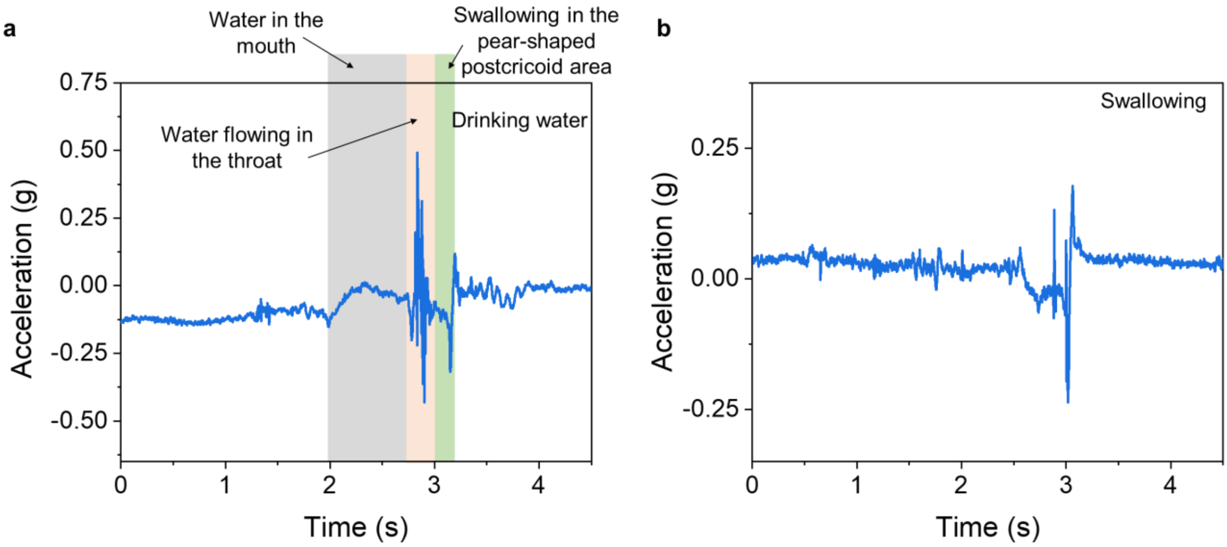

Fig. S22. Comparison of the acceleration responses between a drinking water and **b** swallowing.

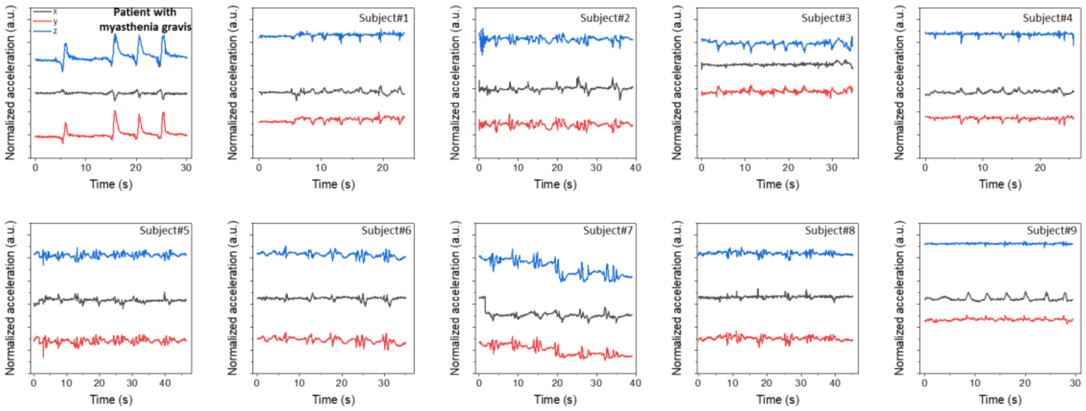

Fig. S23. Normalized swallowing signals of 9 healthy subjects and the patient with myasthenia gravis when they swallows with an repetition.

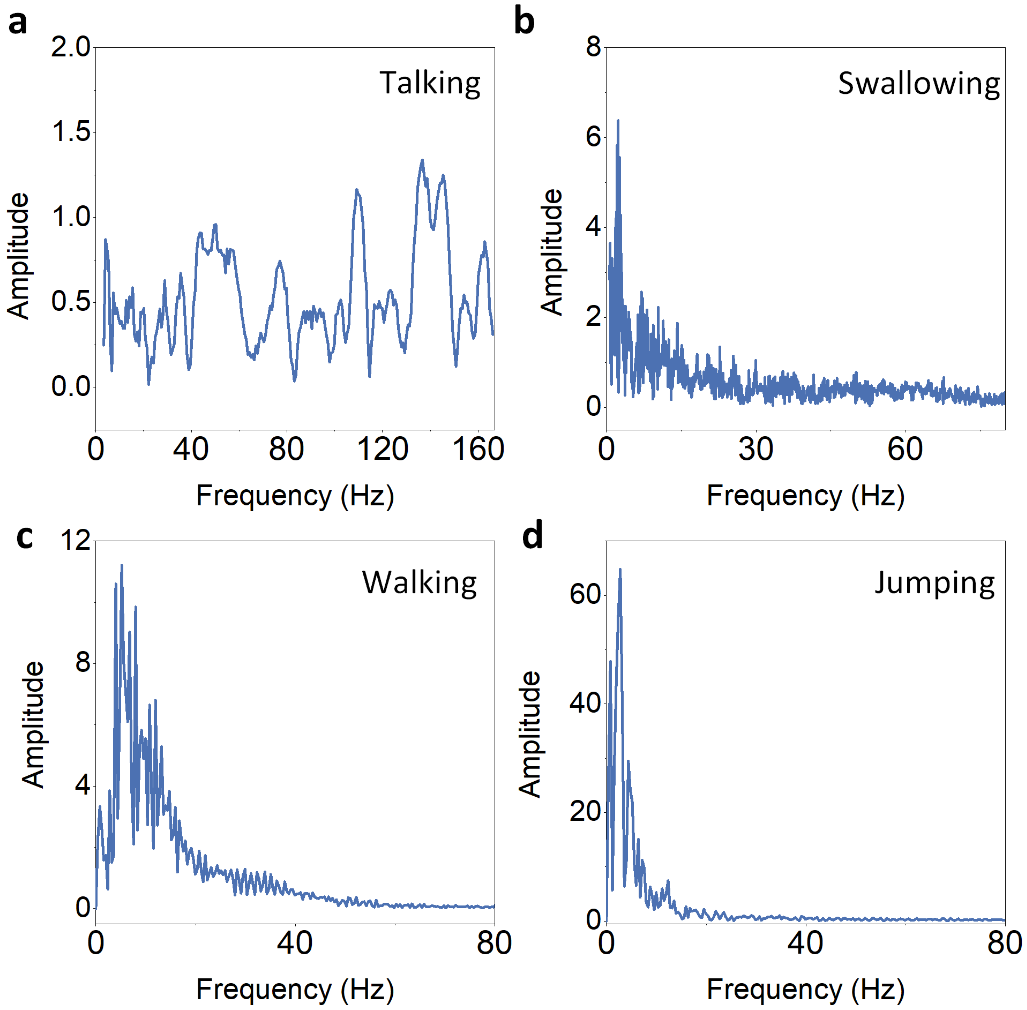

Fig. S24. Fourier transformation of signals captured by the laryngeal patch on a healthy human subject in four typical physiological events: a talking, **b** swallowing, **c** walking, and **d** jumping.

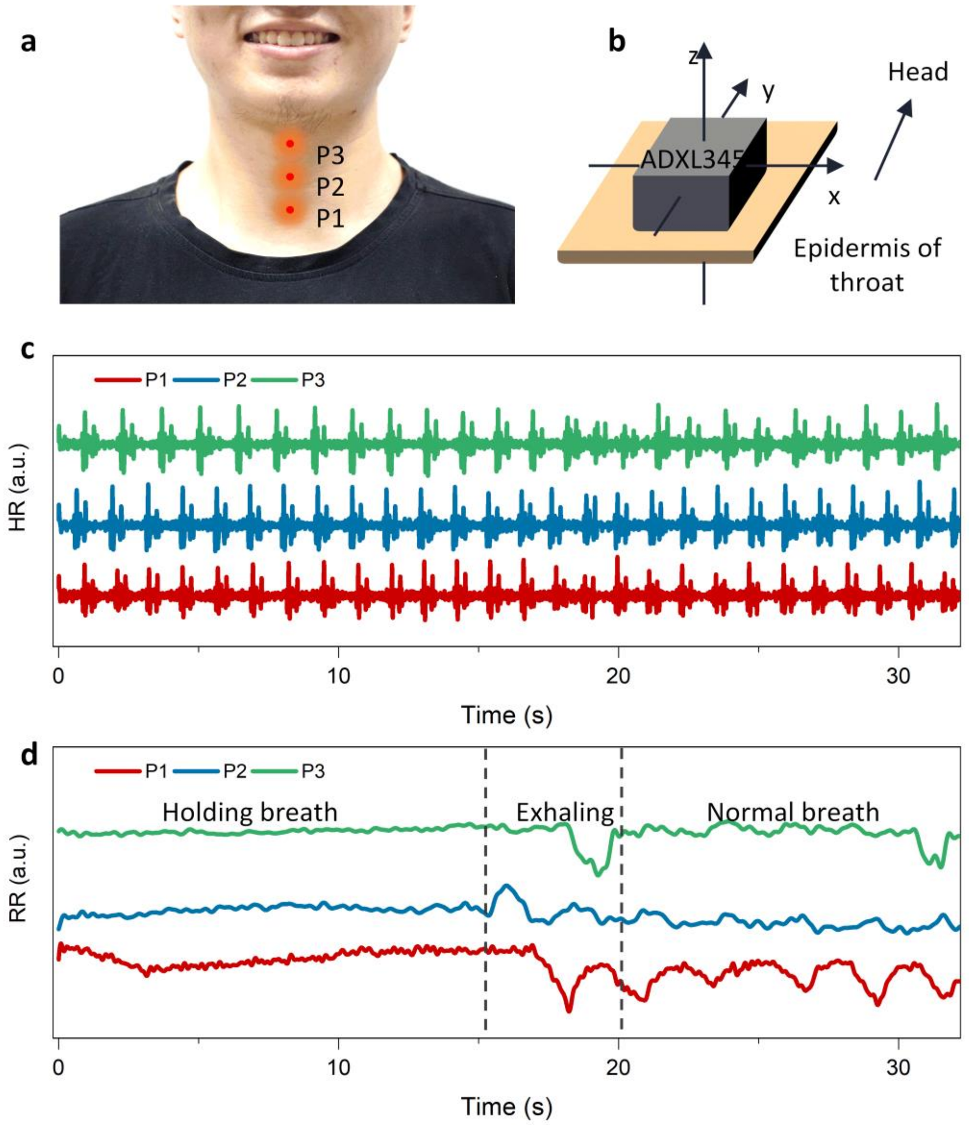

Fig. S25. Decoupled heart rate (HR) and respiration rate (RR) from the acceleration data from the laryngeal patch on varying larynx locations. a Optical image showing the different mounting locations (i.e., P1, P2, and P3 from the bottom to top). **b** Schematic showing the directions of the accelerometer axis relative to the human head direction. **c** Comparison in the real-time HR from three different mounting locations. **d** Continuous monitoring of RR during breath holding, exhaling, and normal breathing.

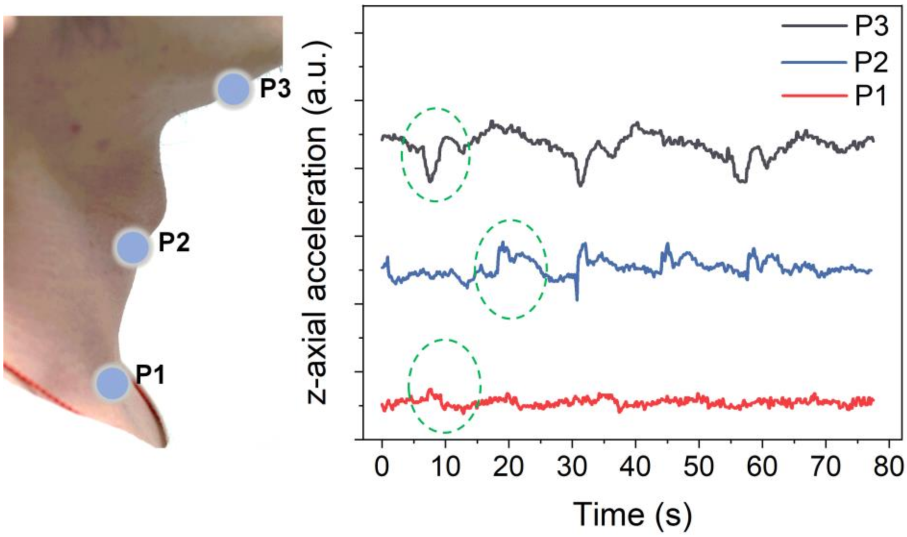

Fig. S26. Three marked positions on a healthy human male for swallowing tests (left) and the signal measured by our integrated device platform (right).

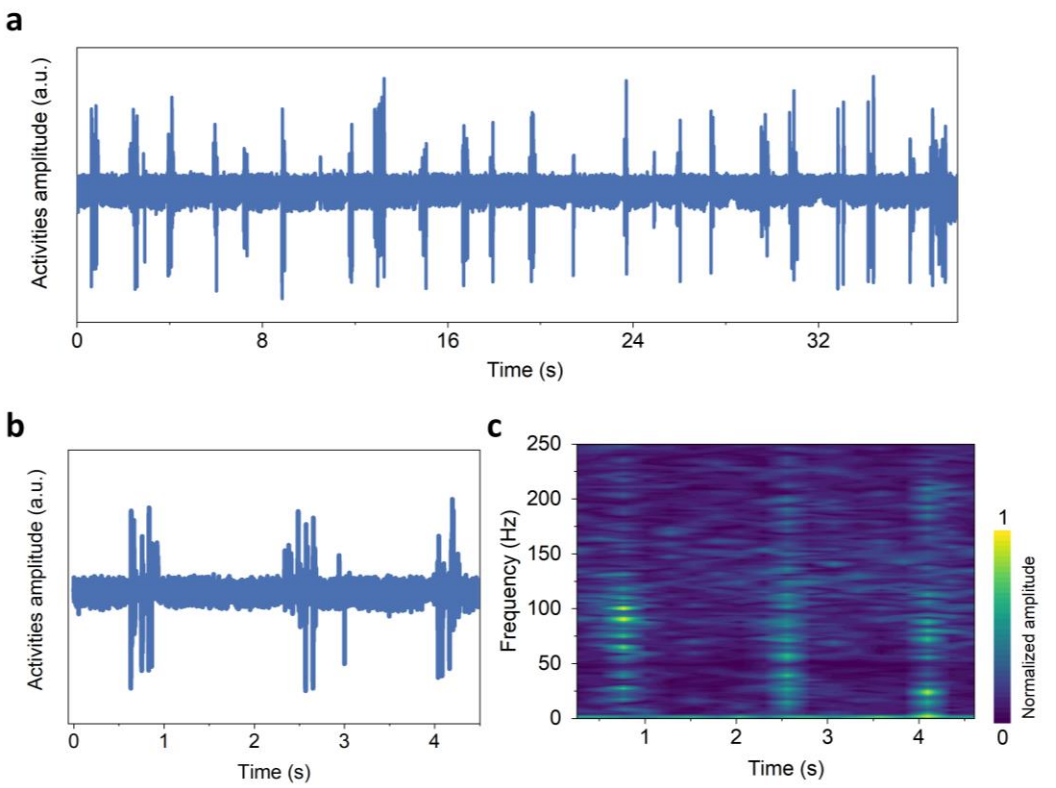

Fig. S27. a sEMG signals over talking captured by the laryngeal patch attached to the middle larynx with the zoomed-in view shown in **b** and **c** corresponding frequency-time spectrum after short-time Fourier transform (STFT).

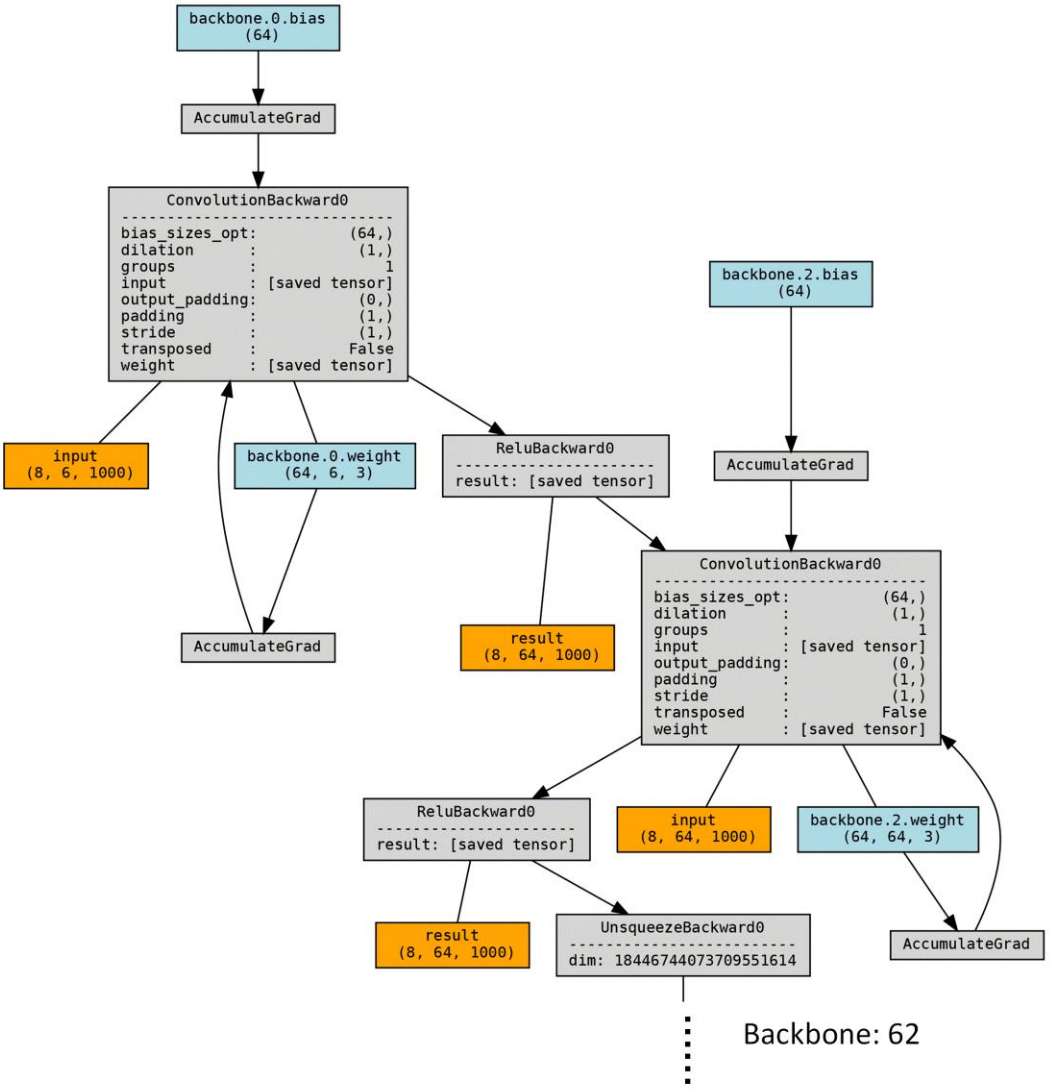

Fig. S28. Flow diagram of the 1-dimension convolutional neural network used for the classification of 13 states in laryngeal postoperative swallowing. The network mainly includes a convolutional layer, active layer, pooling layer, and batch normalization layer.

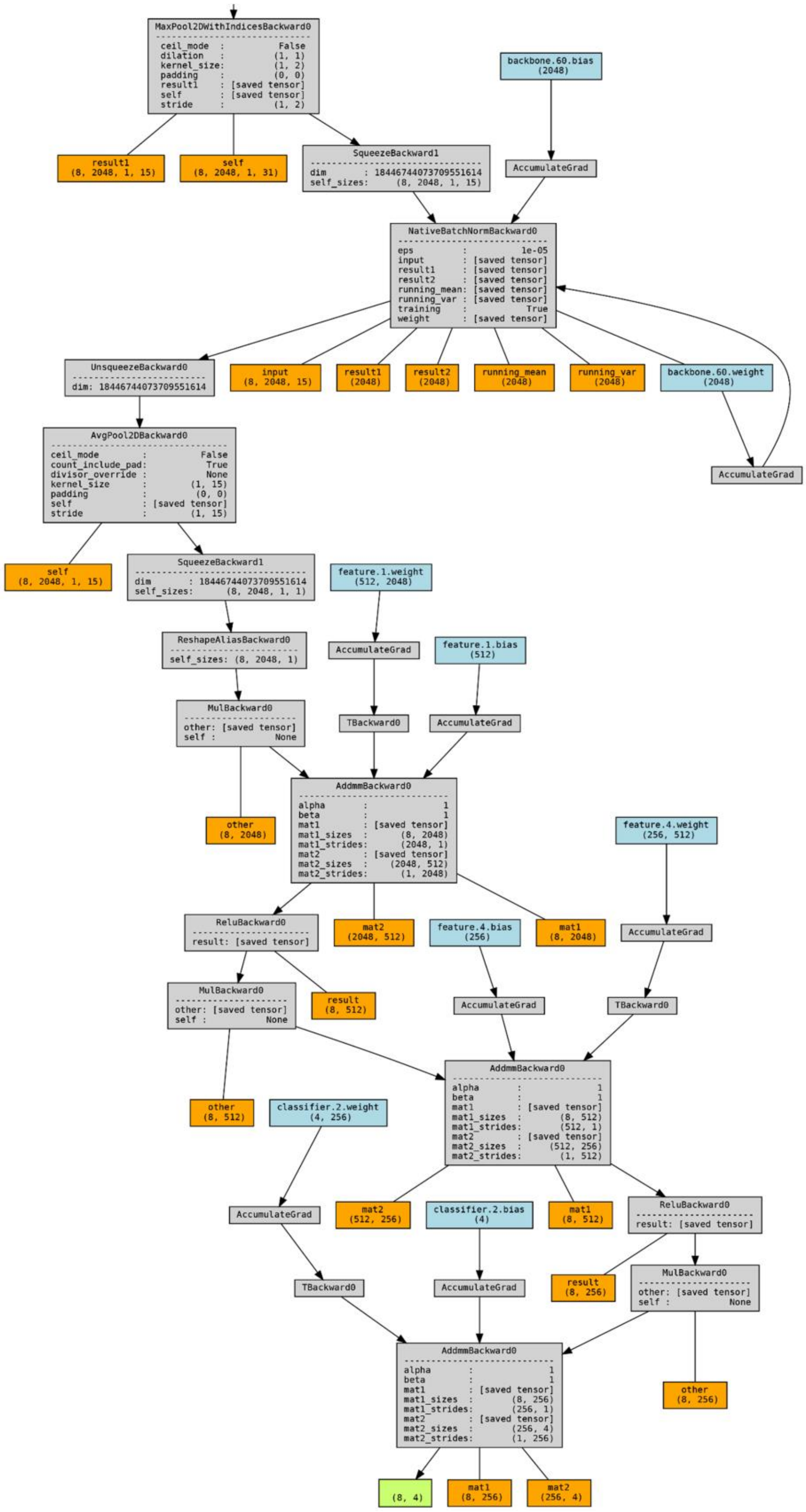

Fig. S29. Flow chart showing the final feature extraction and classification processes that mainly include the convolutional, active, pooling, dropout, batch normalization, max-pooling, and average-pooling layers.

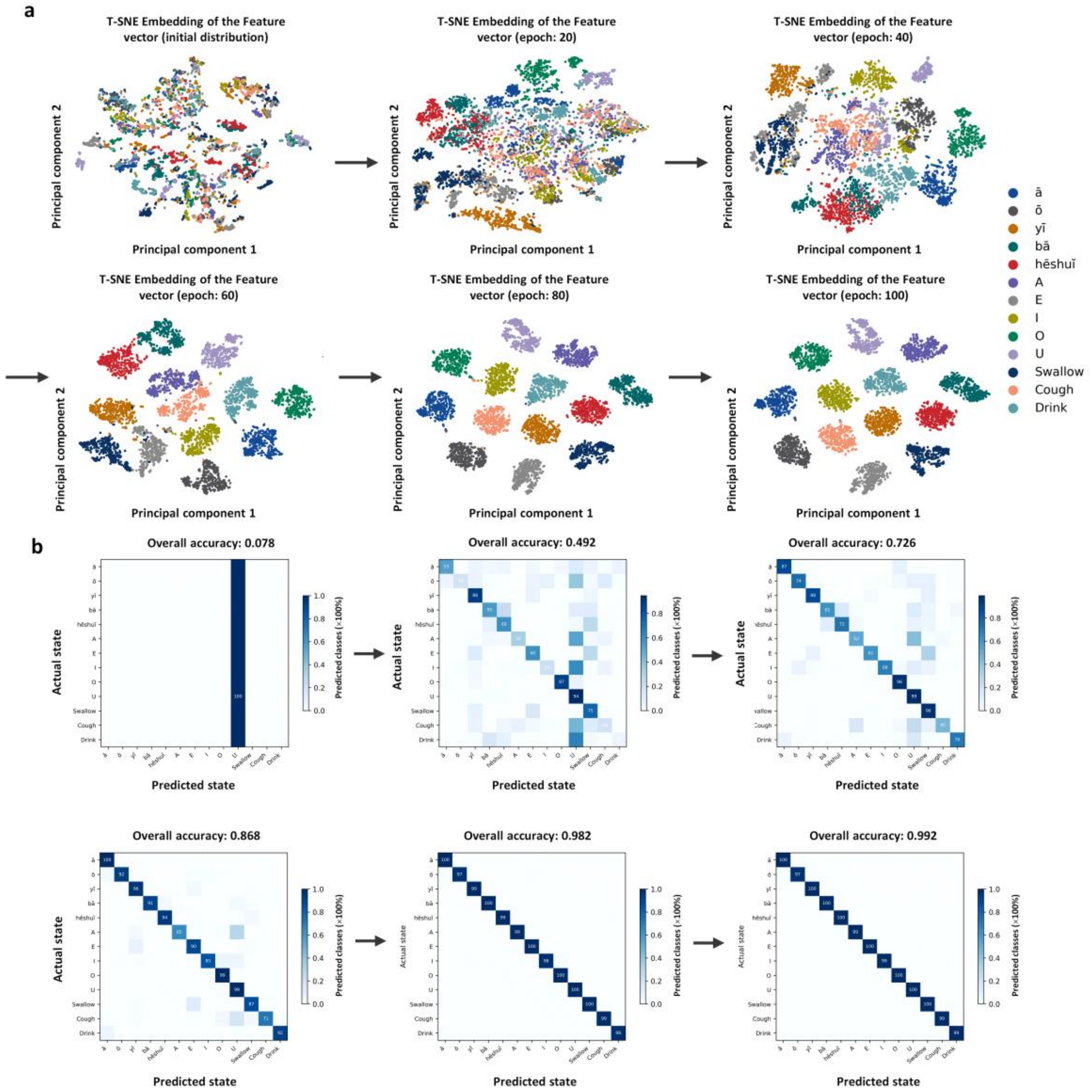

Fig. S30. Feature vector and confusion matrix during the machine learning process for the classification of 13 physiological events. a Feature vector matrix and **b** corresponding confusion matrix from the initial to 100-epoch iterations during t-distributed stochastic neighbor embedding (T-SNE) processing.

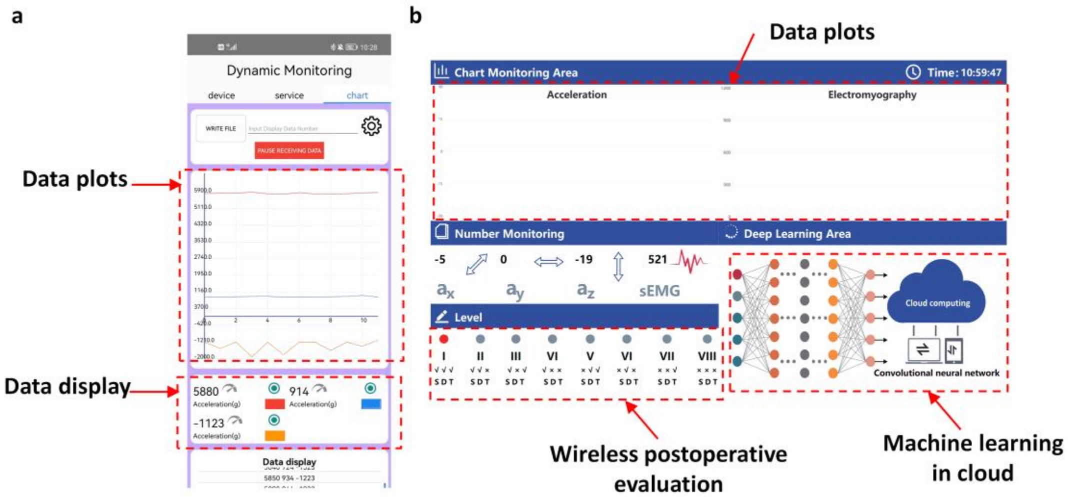

Fig. S31. Display interfaces for continuous real-time monitoring of the signals from the laryngeal patch: a developed APP interface for a smartphone and **b** demonstrated interface in a PC connected to a developed cloud.

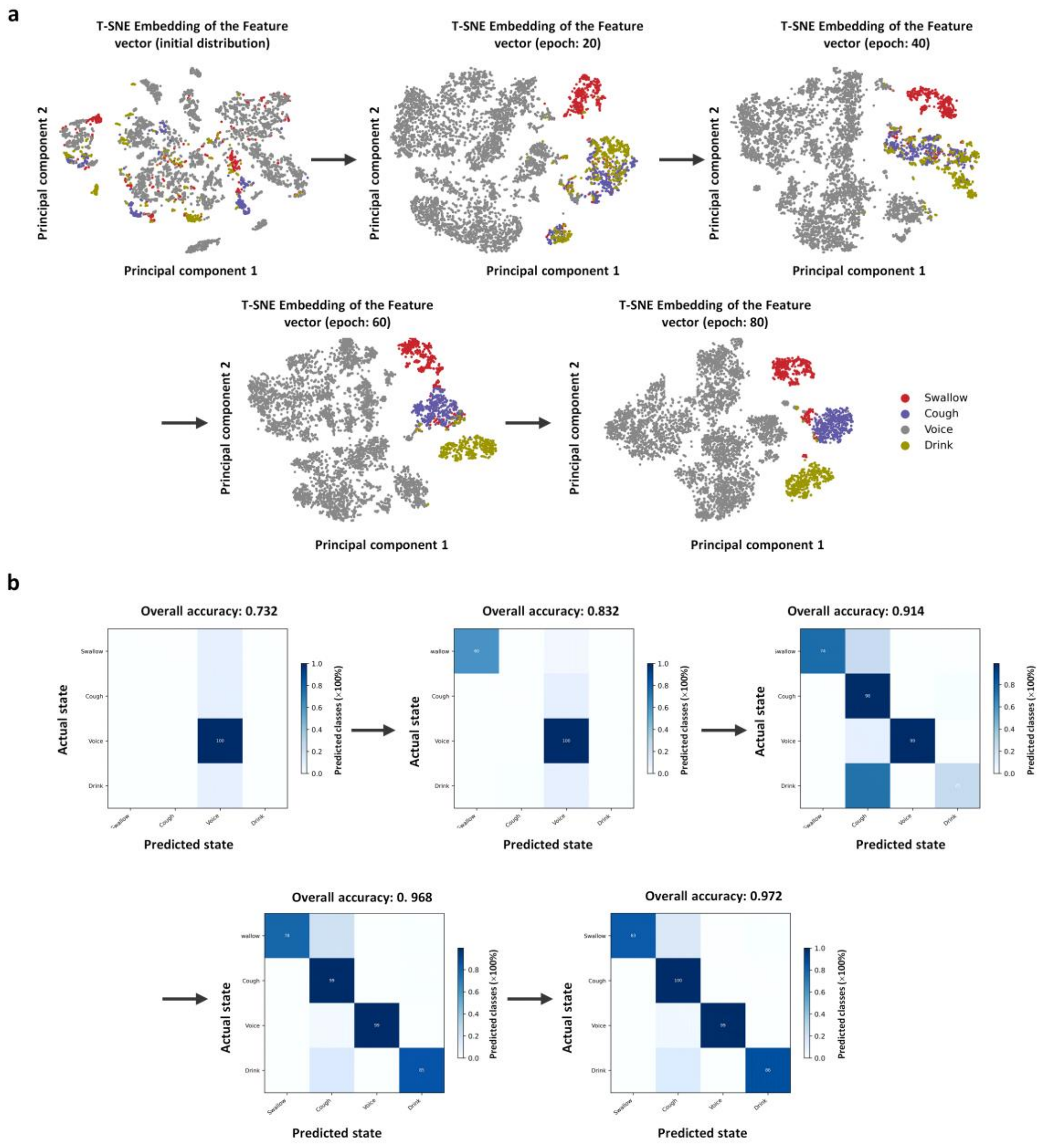

Fig. S32. Feature vector and confusion matrix during the updated machine learning process for the classification of the synthesized 4-category physiological events. a Feature vector matrix and **b** corresponding confusion matrix from the initial to 80-epoch iterations during T-SNE processing.

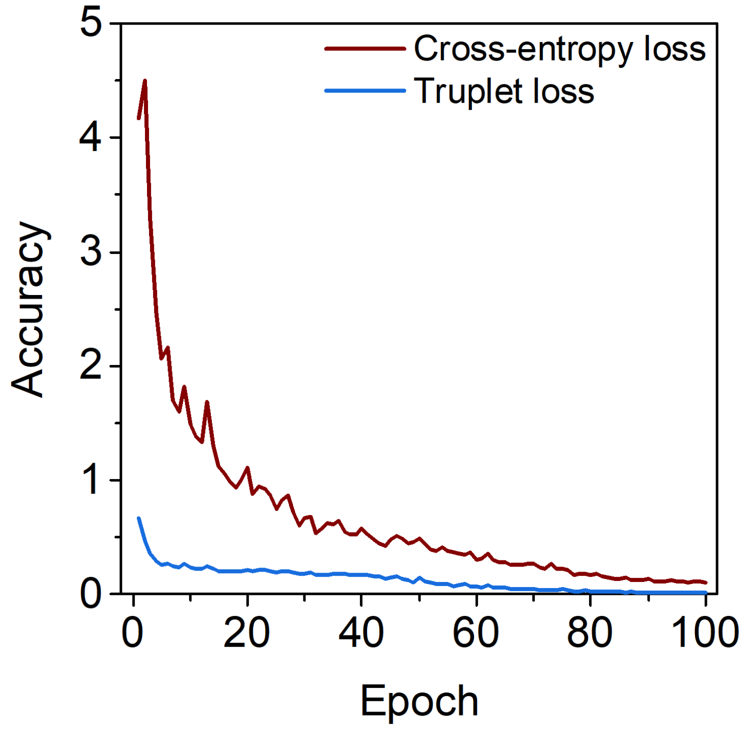

Fig. S33. Normalized loss of the triplet and cross-entropy functions to optimize feature extraction and goal classification for the synthesized 4-category training mode.

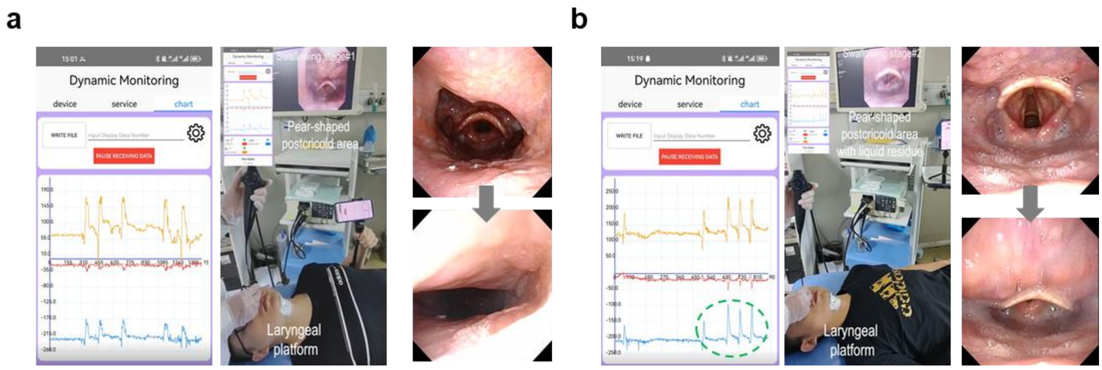

Fig. S34. Comparison between a healthy control and **b** patient with myasthenia gravis during swallowing tests captured by our integrated wireless platform and the FEES: Real-time screenshot on the App interface (left), image showing the device at the oropharyngeal junction area and the FEEs into the esophagus (middle), and FEES images of the closing process of the pear-shaped postcricoid area (right).

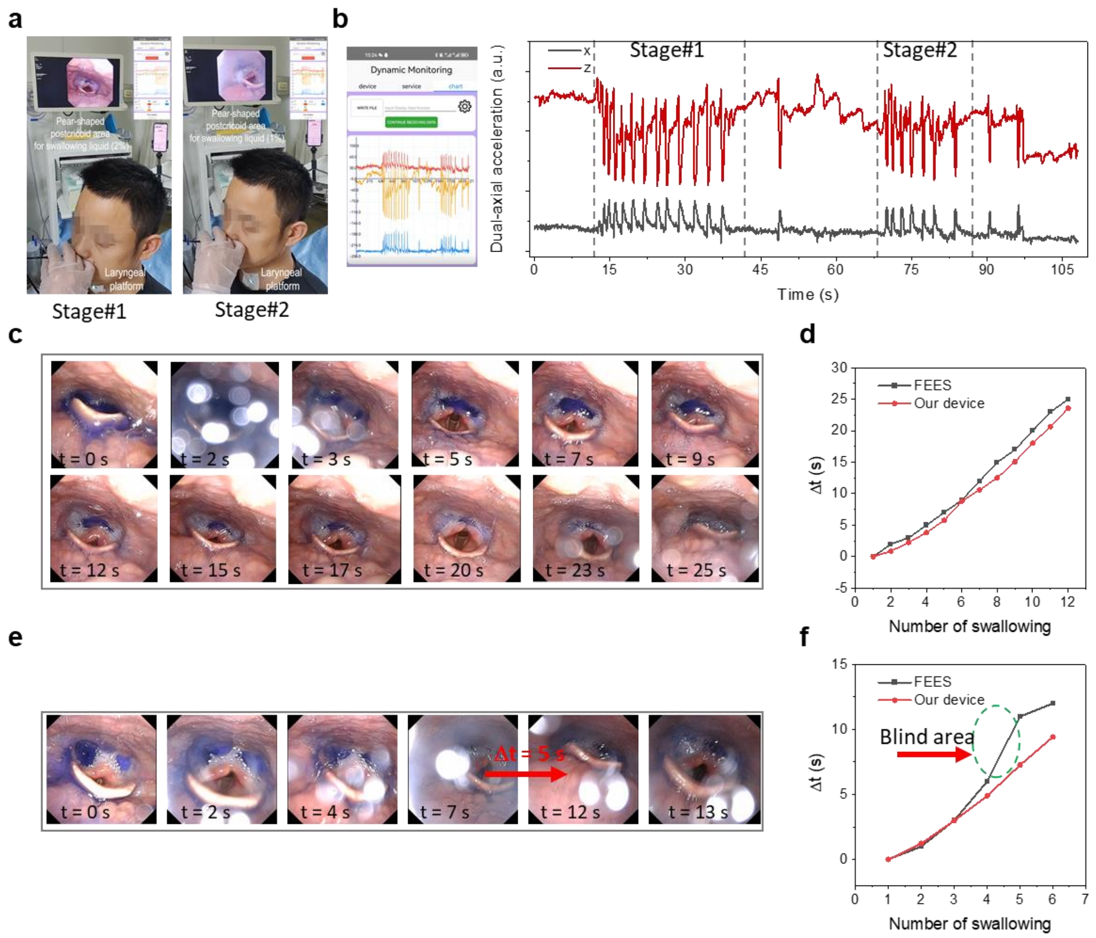

Fig. S35. Volume and viscosity swallowing test with modified safety and effectiveness indicators (VVST-CV) for the patient with myasthenia gravis. a Optical images showing the patient swallowing a blue edible indicator with a weight rate of 2% (left, stage #1) and 1% (right, stage #2). **b** Real-time signals captured by our device during the two-stage swallowing. **c** FEES images in stage #1 and **d** the corresponding time-interval as a function of the number of swallowing. **e** FEES images in stage #2 and **f** the time-interval as a function of number of swallowing.

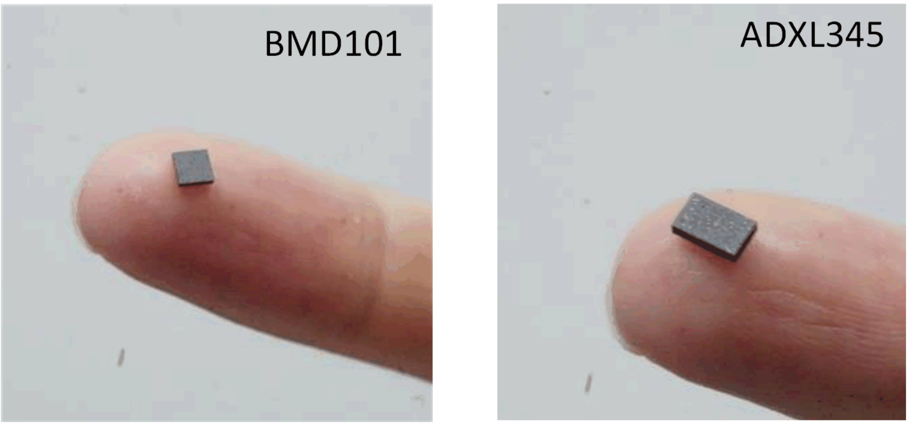

Fig. S36. Optical images of the chips for the acquisition of sEMG (left) and acceleration (right) signals.

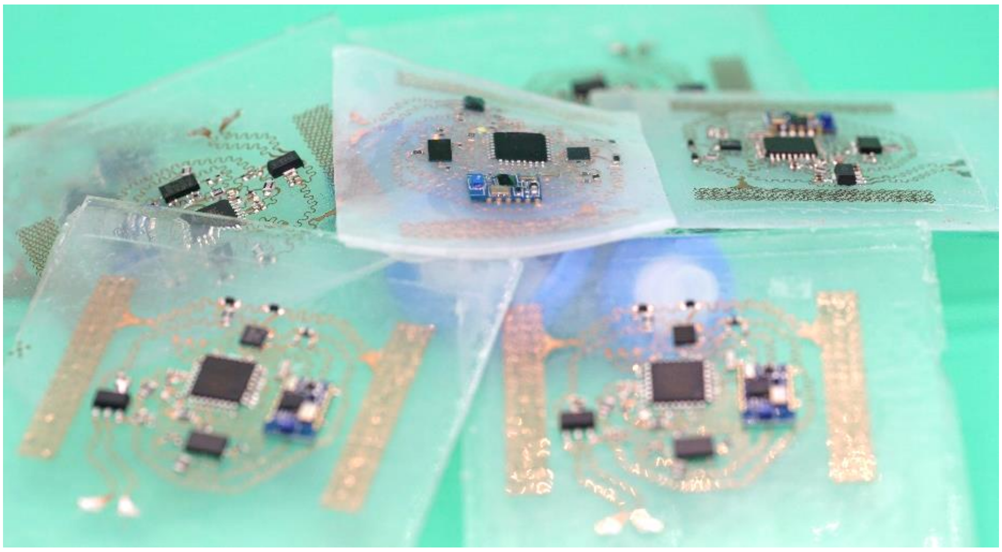

Fig. S37. Optical image of the integrated device platform from batch production.

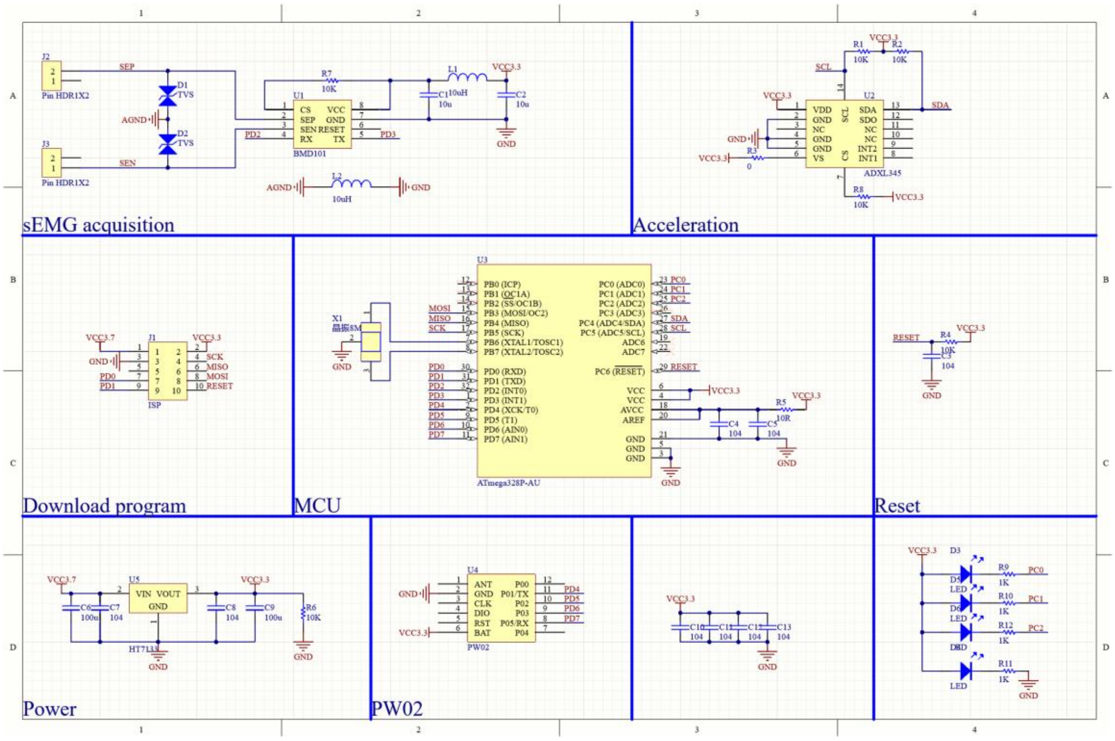

Fig. S38. Circuit diagram of the laryngeal patch.

Table S1 Comparison of different hydrogels (electrodes)

Reference	Conductivity	Transmittance	Biocompatibility	Stretchability	Contact impedance (1 kHz)	Integrated with circuit
27	-	Opaque	good	>100%	>10 kΩ	N
44	0.14 S m^{-1}	98.8%	good	1529%	-	N
45	1 S m^{-1}	98%	good	1000%	10 kΩ	N
46	0.067 S m^{-1}	-	good	2636%	-	N
47	0.019 S m^{-1}	Opaque	good	450%	-	N
48	3.8 S m^{-1}	Opaque	-	3080%	5~6.5 kΩ	N
49	0.91 S m^{-1}	85%	good	105%	14.5 kΩ	N
50	0.25 S m^{-1}	98.8%	good	2900%	31 kΩ	N
51	0.13 S m^{-1}	91%	good	320%	-	N
52	1.2 S m^{-1}	80%	-	850%	-	N
53	0.6 S m^{-1}	-	good	1530%	6.3 kΩ	N
54	0.34 S m^{-1}	95%	-	10000%	-	N
55	0.14 S m^{-1}	-	-	1400%	-	N
56	0.17 S m^{-1}	85%	good	580%	-	N
57	-	>80%	good	150~200%	10-20 kΩ	N
58	47.4 S cm^{-1}	-	good	20%	40 kΩ	N
59	-	<50%	None	None	23.4~38.3 kΩ	N
60	<0.5 S m^{-1}	>80%	good	-	10~20 kΩ	N
This work	**1.7 S m^{-1}**	50%	good	200%	**4.7 kΩ**	√

N no, √ yes, **Integrated with circuit**: integration of the hydrogel with a flexible circuit in the integrated device for health monitoring.

Table S2 Comparison of different laryngeal sensor patches

Sensor patch	Flexibility	Systemic integration	Wireless	Sensing mode	Monitoring	Trained by ML	Reference
Flexible submental sensor	Semi-flexible	N	√	Strain, EMG	Muscle activity, throat movements	N	23
Accelerometer Sensor	Semi-flexible	N	N	Acceleration	Voices	N	24
Mechano-acoustic device	Semi-flexible	√	√	Acceleration	Physiological processes, body motions	N	38
Acoustic MEMS Sensor	/	N	N	Acoustic vibration	Chewing, Swallowing	√	63
Epidermal device	Full-soft	N	N	Acceleration, EMG	Electrophysiological signals	√	64
Dual-axis swallowing accelerometer	Semi-flexible	N	N	Acceleration	Swallowing	N	65
Dual wearable sensor	Semi-flexible	√	√	Acceleration	Artifact-canceled physiological	N	66
Mechano-acoustic sensors	Semi-flexible	√	√	Acceleration	Swallowing, respirations	N	67
Throat vibrator	Rigid	N	N	Microphone vibration	Dysphagia	√	68
Lip-closing force gauge	Rigid	N	N	Force, EMG	Lip-closing	N	16
Neck belt	Semi-flexible	N	N	Displacements	Swallowing	N	69
Acoustic detecting array	Semi-flexible	N	N	Acceleration, microphone	Swallowing sounds	N	70
Hierarchically resistive skins	Full-soft	N	N	Resistive changes	Touch and neck movement	√	71
Wearable artificial throat	Full-soft	N	N	Strain	Voices	√	72
sEMG tattoo	Full-soft	N	N	sEMG	Speech	N	73
2D Metal Film	Full-soft	N	N	Resistive changes	Subvocal talking	N	74
Intelligent artificial throat	Full-soft	N	N	Strain, EMG	Voice signal	√	75
Laser-induced graphene sensor	Semi-flexible	N	N	Resistive changes	Sound signal	N	76
Proposed sensor patch	**Full-soft**	√	√	sEMG, Acceleration	Swallowing, dysphagia	√	This work

N no, √ yes, EMG Electromyogram, sEMG Surface Electromyogram, **ML**: Machine-learning

Table S3 Comparison of the advantage of the integration of two signals with reported methods

Applications	Signal type	Data dimension	Number of Signal	Overall accuracy	Reference
Facial strain and kinematics	Strain	1	1	86.8%	30
Throat activities	Resistance	1	1	92.73%	71
Speech signal	sEMG	1	1	92.3%	73
Speech detection	sEMG	4	1	87.53%	76
Gesture recognition	sEMG	1	1	92.87%	78
Non-invasive identification	sEMG	1	1	92.5%	79
Finger motion	sEMG	4	1	<80%	80
Gesture recognition	Piezoelectric and triboelectric	2	2	82.3%	81
Object recognition	Pressure and temperature	10	2	94%	82
Laryngeal rehabilitation	sEMG and acceleration	**4**	**2**	**98.2%**	This work

sEMG Surface Electromyogram

Table S4 Pathological degree evaluation according to the health condition of three typical event below

Degree \ Behavior	Swallowing ability (S)	Drinking water ability (D)	Talking ability (T)
I	√	√	√
II	√	√	×
III	√	×	√
IV	√	×	×
V	×	√	√
VI	×	√	×
VII	×	×	√
VIII	×	×	×

× Abnormal, √ Normal, Meaning that the condition of the corresponding ability is diagnosed by laryngeal rehabilitative standard during clinical assessments.
