# Supplementary material for: A fully integrated, standalone stretchable device platform with in-sensor adaptive machine learning for rehabilitation
Source: Nat Commun. 2023 Nov 27;14:7769. doi: 10.1038/s41467-023-43664-7 (PMC10682047; doi:10.1038/s41467-023-43664-7)
Supplement: Supplementary file 3 — Description of additional supplementary files [file 41467_2023_43664_MOESM3_ESM.pdf]

Description of additional supplementary files

Supplementary Movie 1 : FEA for the standalone device under stretching, bending, and twisting deformations

Supplementary Movie 2 : Deformationability and wearability of the fabricated device

Supplementary Movie 3 : Real-time robustness tests for the platform under uniaxial 25% stretching

Supplementary Movie 4 : Monitoring for subtle physiological activities at the throat

Supplementary Movie 5 : Monitoring for body motions

Supplementary Movie 6 : Wireless monitoring of overall integrated device

Supplementary Movie 7 : Clinical tests and comparisons
