# Supplementary material for: A fully integrated, standalone stretchable device platform with in-sensor adaptive machine learning for rehabilitation
Source: Nat Commun. 2023 Nov 27;14:7769. doi: 10.1038/s41467-023-43664-7 (PMC10682047; doi:10.1038/s41467-023-43664-7)
Supplement: Supplementary file 11 — Reporting Summary [file 41467_2023_43664_MOESM11_ESM.pdf]

nature portfolio

Corresponding author(s): Libo Gao
Last updated by author(s): Oct 15, 2023

Reporting Summary

Nature Portfolio wishes to improve the reproducibility of the work that we publish. This form provides structure for consistency and transparency in reporting. For further information on Nature Portfolio policies, see our Editorial Policies and the Editorial Policy Checklist.

Statistics

For all statistical analyses, confirm that the following items are present in the figure legend, table legend, main text, or Methods section.

n/a	Confirmed	
☒	☐	The exact sample size (*n*) for each experimental group/condition, given as a discrete number and unit of measurement
☒	☐	A statement on whether measurements were taken from distinct samples or whether the same sample was measured repeatedly
☒	☐	The statistical test(s) used AND whether they are one- or two-sided<br>*Only common tests should be described solely by name; describe more complex techniques in the Methods section.*
☒	☐	A description of all covariates tested
☒	☐	A description of any assumptions or corrections, such as tests of normality and adjustment for multiple comparisons
☒	☐	A full description of the statistical parameters including central tendency (e.g. means) or other basic estimates (e.g. regression coefficient) AND variation (e.g. standard deviation) or associated estimates of uncertainty (e.g. confidence intervals)
☒	☐	For null hypothesis testing, the test statistic (e.g. *F*, *t*, *r*) with confidence intervals, effect sizes, degrees of freedom and *P* value noted<br>*Give P values as exact values whenever suitable.*
☒	☐	For Bayesian analysis, information on the choice of priors and Markov chain Monte Carlo settings
☒	☐	For hierarchical and complex designs, identification of the appropriate level for tests and full reporting of outcomes
☒	☐	Estimates of effect sizes (e.g. Cohen's *d*, Pearson's *r*), indicating how they were calculated

Our web collection on statistics for biologists contains articles on many of the points above.

Software and code

Policy information about availability of computer code

Data collection	Origin2023, Microsoft excel, Arduino IDE.
Data analysis	Python, Pycharm Community Edition 2022.2.1

For manuscripts utilizing custom algorithms or software that are central to the research but not yet described in published literature, software must be made available to editors and reviewers. We strongly encourage code deposition in a community repository (e.g. GitHub). See the Nature Portfolio guidelines for submitting code & software for further information.

Data

Policy information about availability of data

All manuscripts must include a data availability statement. This statement should provide the following information, where applicable:

- Accession codes, unique identifiers, or web links for publicly available datasets
- A description of any restrictions on data availability
- For clinical datasets or third party data, please ensure that the statement adheres to our policy

Source data are provided with this paper and its supplementary information.

Research involving human participants, their data, or biological material

Policy information about studies with human participants or human data. See also policy information about sex, gender (identity/presentation), and sexual orientation and race, ethnicity and racism.

Reporting on sex and gender	There are 18 participants in this experiment (including fourteen healthy human subjects and two patients (one with myasthenia gravis and the other with the laryngeal cancer). The sex and gender were not considered in the study design. There are no self-selection biases or other biases on sex or gender.
Reporting on race, ethnicity, or other socially relevant groupings	There are no any relevant grouping experiments for race, ethnicity, or other socially.
Population characteristics	All participants are over 18 years old. In addition, one patient were diagnosed the myasthenia gravis by the Tangdu hospital (Air Force Medical University) and another just suffered the laryngeal cancer CHEP operation during the testing.
Recruitment	All participants are our colleague in the lab. We introduce and explain the significance of the experiment within the laboratory, and all participants voluntarily participate in the registration process
Ethics oversight	All human subject studies were approved by the Institutional Review Board of the First Affiliated Hospital of the Air Force Medical University (protocol: KY20222259-C-1), and the volunteers gave informed consent.

Note that full information on the approval of the study protocol must also be provided in the manuscript.

Field-specific reporting

Please select the one below that is the best fit for your research. If you are not sure, read the appropriate sections before making your selection.

☒ Life sciences ☐ Behavioural & social sciences ☐ Ecological, evolutionary & environmental sciences

For a reference copy of the document with all sections, see nature.com/documents/nr-reporting-summary-flat.pdf

Life sciences study design

All studies must disclose on these points even when the disclosure is negative.

Sample size	No sample size calculation was performed. A sample size of 3 provides sufficient information on the repeatability of different types of various signals.
Data exclusions	No data were excluded from the analyses.
Replication	Verification of the replication of the cycle test more than three times. The results were were replicable, and any small discrepancy in the data ere displayed explicitly.
Randomization	All devices tested were selected randomly.
Blinding	The investigators were blinded to group allocation during data collection and analysis.

Reporting for specific materials, systems and methods

We require information from authors about some types of materials, experimental systems and methods used in many studies. Here, indicate whether each material, system or method listed is relevant to your study. If you are not sure if a list item applies to your research, read the appropriate section before selecting a response.

Materials & experimental systems

n/a	Involved in the study	
☒	☐	Antibodies
☒	☐	Eukaryotic cell lines
☒	☐	Palaeontology and archaeology
☒	☐	Animals and other organisms
☐	☒	Clinical data
☒	☐	Dual use research of concern
☒	☐	Plants

Methods

n/a	Involved in the study	
☒	☐	ChIP-seq
☒	☐	Flow cytometry
☒	☐	MRI-based neuroimaging

Clinical data

Policy information about clinical studies

All manuscripts should comply with the ICMJEguidelines for publication of clinical research and a completedCONSORT checklist must be included with all submissions.

Clinical trial registration	KY20222259-C-1
Study protocol	By comparing the changes in the vibration signals of different people' throat during speeching, swallowing, coughing, and drinking water, the designed sensor are optimized to provide technical assistance for swallowing patients.
Data collection	Continuous testing is required for the vibration signal measurement of 18 subjects during the speeching, swallowing, coughing, and drinking water. Each subject performs the same action 60 times and the data were recorded by the patch-sensor wirelessly.
Outcomes	N/A

Plants

Seed stocks	N/A
Novel plant genotypes	N/A
Authentication	N/A
